# Supplementary material for: The interactome of intact mitochondria by cross-linking mass spectrometry provides evidence for coexisting respiratory supercomplexes
Source: Mol Cell Proteomics. 2017 Dec 8;17(2):216–32. doi: 10.1074/mcp.RA117.000470 (PMC5795388; doi:10.1074/mcp.RA117.000470)
Supplement: Supplemental Data [file supp_RA117.000470_133922_0_supp_23978_fzffwf.zip › spectra_annotation/mito_spectra_annotation/180-1-12-1-6-1.pdf]

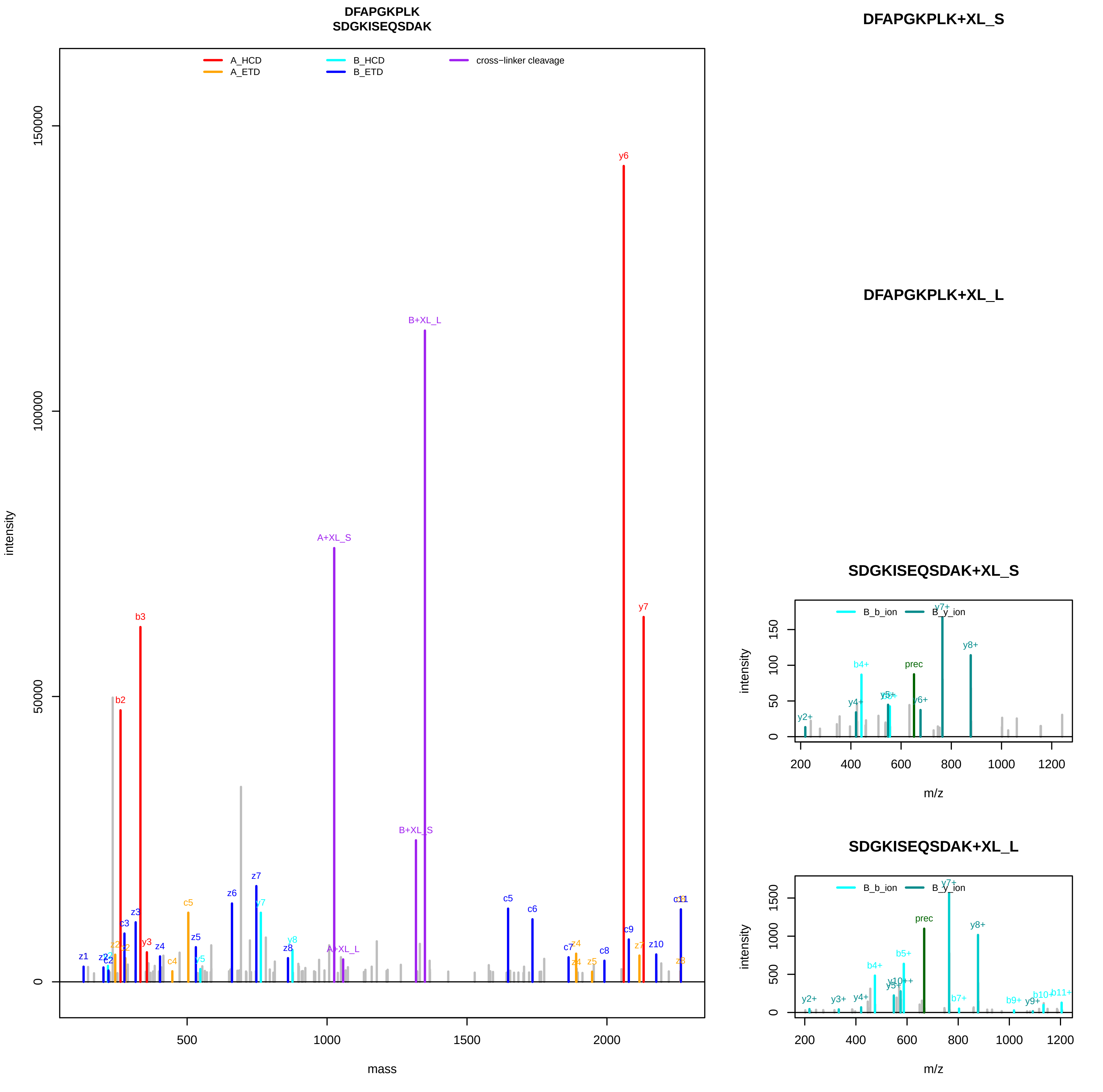
DFAPGKPLK
SDGKISEQSDAK
A_HCD
A_ETD
B_HCD
B_ETD
cross−linker cleavage
intensity
mass
0
50000
100000
150000
500
1000
1500
2000
y6
B+XL_L
A+XL_S
y7
b3
b2
B+XL_S
A+XL_L
z1
c2
c3
z3
y3
z4
c4
c5
z5
y5
z6
z7
y7
y8
z8
c5
c6
c7
z4
c8
c9
z10
c11
z5
z7
DFAPGKPLK+XL_S
DFAPGKPLK+XL_L
SDGKISEQSDAK+XL_S
B_b_ion
B_y_ion
y7+
y8+
b4+
prec
y4+
y5+
y6+
y2+
intensity
m/z
0
50
100
150
200
400
600
800
1000
1200
SDGKISEQSDAK+XL_L
B_b_ion
B_y_ion
y7+
prec
y8+
b5+
b4+
y10++
y5+
y2+
y3+
y4+
b7+
b9+
y9+
b10+
b11+
intensity
m/z
0
500
1000
1500
200
400
600
800
1000
1200
